# Development and Validation of a Machine Learning Model for Early Prediction of Acute Kidney Injury in Neurocritical Care: A Comparative Analysis of XGBoost, GBM, and Random Forest Algorithms

**DOI:** 10.3390/diagnostics15162061

**Published:** 2025-08-17

**Authors:** Keun Soo Kim, Tae Jin Yoon, Joonghyun Ahn, Jeong-Am Ryu

**Affiliations:** 1 Department of Emergency Medicine, Uijeongbu Eulji Medical Center, Eulji University, Uijeongbu 11749, Republic of Korea; mdsolt@gmail.com; 2 Department of Emergency Medicine, Bucheon Sejong Hospital, Bucheon 14754, Republic of Korea; zangtaejin@naver.com; 3 Biomedical Statistics Center, Data Science Research Institute, Samsung Medical Center, Seoul 06351, Republic of Korea; jhguy.ahn@samsung.com; 4 Department of Critical Care Medicine, Samsung Medical Center, School of Medicine, Sungkyunkwan University, Seoul 06351, Republic of Korea; 5 Department of Neurosurgery, Samsung Medical Center, School of Medicine, Sungkyunkwan University, Seoul 06351, Republic of Korea

**Keywords:** acute kidney injury, neurocritical care, hyperosmolar therapy, machine learning, biomarkers

## Abstract

**Background:** Acute Kidney Injury (AKI) is a pivotal concern in neurocritical care, impacting patient survival and quality of life. This study harnesses machine learning (ML) techniques to predict the occurrence of AKI in patients receiving hyperosmolar therapy, aiming to optimize patient outcomes in neurocritical settings. **Methods:** We conducted a retrospective cohort study of 4886 patients who underwent hyperosmolar therapy in the neurosurgical intensive care unit (ICU). Comparative predictive analyses were carried out using advanced ML algorithms—eXtreme Gradient Boosting (XGBoost), Gradient Boosting Machine (GBM), Random Forest (RF)—against standard multivariate logistic regression. Predictive performance was assessed using an 8:2 training-testing data split, with model fine-tuning through cross-validation. **Results:** The RF with KNN imputation showed slightly better performance than other approaches in predicting AKI. When applied to an independent test set, it achieved a sensitivity of 79% (95% CI: 70–87%) and specificity of 85% (95% CI: 82–88%), with an overall accuracy of 84% (95% CI: 81–87%) and AUROC of 0.86 (95% CI: 0.82–0.91). The multivariate logistic regression analysis, while informative, showed less predictive strength compared to the ML models. Delta chloride levels and serum osmolality proved to be the most influential predictors, with additional significant variables including pH, age, bicarbonate, and the osmolar gap. **Conclusions:** The prominence of delta chloride and serum osmolality among the predictive variables underscores its potential as a biomarker for AKI risk in this patient population.

## 1. Background

Acute kidney injury (AKI) is a crucial determinant of prognosis in critically ill patients, significantly impacting mortality and the length of hospital stays in the intensive care unit (ICU) [1,2]. The occurrence of AKI, particularly in conjunction with other organ dysfunctions, escalates the mortality rate substantially, often reaching 60 to 80% [3]. This underlines the critical importance of early identification and management of AKI in the ICU setting.

In neurocritically ill patients, AKI emerges as a particularly critical issue due to unique pathophysiological challenges and treatment modalities [4,5]. These patients frequently require hyperosmolar therapy, including mannitol and hypertonic saline, for managing cerebral edema following severe neurological injuries. While these therapies are crucial for reducing intracranial pressure, they have been consistently linked to an increased incidence of AKI [4,5,6,7,8]. Studies have shown that AKI affects 18–46% of neurocritical care patients, with significantly higher mortality rates and longer ICU stays compared to those without AKI [7,8].

Traditional AKI prediction models have predominantly relied on clinical scoring systems and logistic regression approaches incorporating established risk factors such as baseline creatinine, comorbidities, and severity scores [6,9]. However, these conventional models demonstrate significant limitations when applied to neurocritical care populations. They often lack specificity for the unique brain-kidney interactions and inadequately account for the complex effects of hyperosmolar therapy on renal function [10,11]. Furthermore, traditional statistical approaches may fail to capture non-linear relationships and complex interactions between multiple variables that characterize AKI development in this setting [12,13].

Recent studies have highlighted the importance of previously underrecognized risk factors, particularly electrolyte disturbances such as hyperchloremia and acid-base imbalances, in patients receiving hyperosmolar therapy [14,15]. However, comprehensive studies specifically examining AKI prediction in neurocritical care populations using advanced analytical approaches remain limited [16,17].

Machine learning (ML) techniques have emerged as promising tools for improving clinical prediction models, excelling at identifying complex patterns in high-dimensional datasets that traditional statistical methods might miss [18,19,20,21,22,23,24]. Several studies have demonstrated the superior performance of ML approaches compared to conventional methods in various AKI prediction scenarios [25,26]. However, most existing ML-based AKI prediction models have been developed for general ICU populations, with limited focus on neurocritical care settings receiving hyperosmolar therapy [27,28].

In light of these challenges, our study employed ML techniques to predict AKI in neurocritically ill patients undergoing hyperosmolar therapy. Utilizing historical patient data and sophisticated algorithmic models, ML can offer a novel approach to uncover complex interactions and risk factors not easily identifiable through traditional analysis [18,19,20,21,22,23,24]. Our goal is to harness ML to offer insights that could significantly improve patient management in neurocritical care settings.

## 2. Literature Review

AKI is a crucial determinant of prognosis in critically ill patients, significantly impacting mortality and ICU length of stay [1,2]. In neurocritically ill patients, AKI emerges as a particularly critical issue affecting 18–46% of patients, with mortality rates reaching 60–80% [3,7,8]. These patients frequently require hyperosmolar therapy for managing cerebral edema, which paradoxically increases AKI risk despite being essential for reducing intracranial pressure [4,5,6,7,8].

Traditional AKI prediction models have relied on clinical scoring systems and logistic regression incorporating established risk factors such as baseline creatinine and severity scores [6,9]. However, these conventional models demonstrate significant limitations in neurocritical care populations, lacking specificity for brain-kidney interactions and inadequately accounting for hyperosmolar therapy effects [10,11]. Recent studies have highlighted previously underrecognized risk factors, particularly electrolyte disturbances such as hyperchloremia, though comprehensive studies using advanced analytical approaches remain limited [14,15,16,17]. ML techniques have emerged as promising tools for improving clinical prediction models, demonstrating superior performance compared to conventional methods in various AKI prediction scenarios [18,19,20,21,22,23,24]. However, most existing ML-based models have been developed for general ICU populations, with limited focus on neurocritical care settings [27,28]. Existing literature reveals several studies addressing AKI prediction in neurocritical care, though significant gaps remain (Table 1). Studies by Buttner et al. and others have achieved modest performance (AUC 0.69–0.78) using traditional approaches, but were limited by small sample sizes, single-center designs, and reliance on conventional statistical methods [7,14,16,29,30,31,32]. This comprehensive review identifies critical needs for larger cohorts, systematic ML algorithm comparison, and novel biomarker exploration in neurocritical care AKI prediction.

This table summarizes existing studies on AKI prediction in neurocritical care and general ICU populations to highlight research gaps that our study addresses. The table includes studies focusing on neurocritical care patients, general ICU populations using ML approaches, and studies specifically examining hyperosmolar therapy-related AKI. Key limitations identified include small sample sizes in neurocritical care studies, limited exploration of ML algorithms in this population, insufficient focus on hyperosmolar therapy-induced AKI, and lack of comprehensive biomarker analysis, including novel predictors such as delta chloride levels. Our study addresses these gaps through a large-scale cohort (*n* = 4886), systematic comparison of multiple ML algorithms, and identification of delta chloride as a novel predictive biomarker in neurocritical care patients receiving hyperosmolar therapy.

This comprehensive review reveals several critical gaps in the existing literature. First, most studies focusing specifically on neurocritical care populations have employed relatively small sample sizes, limiting the generalizability and statistical power of their findings. Second, while ML approaches have shown promise in general ICU populations, their systematic application and comparison in neurocritical care settings remain underexplored. Third, although several studies have identified the importance of electrolyte disturbances, particularly hyperchloremia, a comprehensive investigation of novel biomarkers such as delta chloride levels has been limited. Finally, most existing research has focused on specific neurological conditions rather than the broader neurocritical care population receiving hyperosmolar therapy.

## 3. Study Contributions and Objectives

Building upon the identified gaps in existing literature, this study makes several key contributions to the field of AKI prediction in neurocritical care. We present the largest single-center cohort of neurocritical care patients receiving hyperosmolar therapy (*n* = 4886), providing substantially greater statistical power than previous studies. Additionally, we systematically evaluate seven ML algorithms specifically optimized for this population, filling a critical gap in advanced analytics applications. Most importantly, we identify and validate delta chloride as a novel, clinically actionable biomarker that advances beyond traditional static chloride measures to capture dynamic changes over time.

Our primary objective is to develop and validate superior ML-based prediction models for early AKI detection in neurocritical care settings. Specifically, we aim to compare ML algorithms against traditional approaches, identify the most relevant predictors of AKI in patients receiving hyperosmolar therapy, and develop a robust, clinically implementable model with optimal performance metrics. Through comprehensive validation and feature importance analysis, we seek to provide both practical clinical tools and new insights into hyperosmolar therapy-induced AKI pathophysiology.

## 4. Methods

### 4.1. Study Population

This retrospective observational study was carried out at a single center, the Samsung Medical Center, a tertiary hospital in Seoul, Republic of Korea. It encompassed adult patients admitted to the neurosurgical ICU from January 2013 to September 2019. The Institutional Review Board (IRB) of Samsung Medical Center approved the study (approval number SMC 2020-09-082), and due to its retrospective nature, the requirement for informed consent was waived by the IRB. The study’s criteria included patients admitted to the neurosurgical ICU who received hyperosmolar therapy and had comprehensive initial laboratory tests—serum sodium, chloride, osmolality, urea, glucose, creatinine, glomerular filtration rate (GFR), and arterial blood gas analysis—performed within the first 12 h following ICU admission, along with subsequent follow-up levels. Exclusion criteria were patients below 18 years, those with end-stage renal disease on renal replacement therapy, those with inadequate medical records, a ‘do not resuscitate’ order, admissions to departments other than neurosurgery, transfers to other hospitals, or an uncertain prognosis.

### 4.2. Definitions and Outcomes

In this study, we collected baseline characteristics such as comorbidities, behavioral risk factors, ICU management, and laboratory data retrospectively using our center’s “Clinical Data Warehouse Darwin-C”. This data warehouse was designed specifically for investigators to search and retrieve de-identified medical records from electronic archives. Laboratory data were collected at least once daily from all neurosurgical patients admitted to the ICU. To assess kidney function, we used the Modification of Diet in Renal Disease 4-variable formula to calculate the GFR [33]. Perioperative acute renal dysfunction was defined according to the Acute Kidney Injury Network (AKIN) criteria [34]. “Stage 1” was defined as an increase of creatinine by ≥0.3 mg/dL or 1.5–1.9 times, “stage 2” as an increase of creatinine 2.0–2.9 times, and “stage 3” as an increase of creatinine 3 times [34]. We calculated osmolality (mOsm/kg) using the following formula: serum sodium level (mEq/L) × 2 + (glucose [mg/dL]/18) + (blood urea nitrogen [mg/dL]/2.3) [35]. The difference between the measured osmolarity and the calculated osmolality was defined as the osmolar gap (OG) [35]. End-stage renal disease was defined as being on dialysis or having a GFR < 30 mL/min/1.73 m^2^ for at least 12 weeks [36]. Delta chloride was defined as the difference between the initial chloride level and the highest chloride level observed during the patient’s stay in the ICU. Sodium level, serum osmolality, and chloride level were defined as the highest values recorded during the patient’s ICU stay. On the other hand, pH and bicarbonate level were defined as the lowest values recorded during the same period. The primary endpoint of this study was the occurrence of AKI, which was defined as a ≥0.3-mg/dL increase in serum creatinine level above baseline or a change of ≥50%, according to the AKIN criteria (Stage 1, 2, or 3) [2].

### 4.3. ML Models

In this study, 7 ML algorithms were developed and evaluated for their ability to predict the occurrence of AKI in a binary classification task. These algorithms included random forest (RF), AdaBoost Classification Trees (AdaBoost), Bagged CART (Bagging), Stochastic Gradient Boosting (GBM), eXtreme Gradient Boosting (XGBoost), Multivariate Adaptive Regression Spline (MARS), and Support Vector Machines with Radial Basis Function Kernel (SVM). Univariate analysis was performed to select the most relevant variables for model development. To ensure that the imbalanced outcome variable (AKI: 16.6% vs. non-AKI:83.4%) was equally represented across data subsets, we split the dataset into training (80%) and testing (20%) sets using stratified partitioning. During model training and hyperparameter optimization, stratified 10-fold cross-validation was employed using AKI status as the stratification variable. Final model performance was evaluated exclusively on the independent testing set (20% holdout) that remained completely separate from any training or hyperparameter tuning processes [37,38,39]. To address missing values in the dataset, two preprocessing strategies were employed: exclusion of incomplete cases and k-nearest neighbors (KNN) imputation. These approaches were compared in the development of ML models to assess their predictive performance. Data preprocessing included scaling of continuous variables and one-hot encoding of categorical variables (such as cause of ICU admission) to convert them into binary indicator variables for each category level, enabling proper incorporation into ML algorithms. For the target variable, we employed a binary classification approach with patients classified as either “AKI” or “non-AKI” based on the AKIN criteria, where AKI was defined as meeting any stage of the AKIN criteria (≥0.3 mg/dL increase in serum creatinine above baseline or ≥50% change), coded as 1 for AKI and 0 for non-AKI. Each ML algorithm was trained with its respective optimal hyperparameters until convergence on the training set. To address the class imbalance during model training, we additionally applied synthetic oversampling techniques, including SMOTE (Synthetic Minority Oversampling Technique) and ROSE (Random Over-Sampling Examples), using the trainControl function in the caret package. These methods were used to assess their impact on classifier performance during cross-validation within the training data. Separately, for diagnostic performance evaluation on the independent validation set, we employed optimal threshold selection strategies such as the closest-to-top-left method on ROC analysis. This allowed us to determine the best cutoff values for binary classification and to report sensitivity, specificity, accuracy, and AUROC based on clinically relevant thresholds. We systematically optimized hyperparameters via grid search combined with 10-fold cross-validation, using AUROC as the objective function. For each algorithm, a comprehensive tuning grid was defined to explore key hyperparameter combinations. The final model performance was then evaluated on an independent holdout test set (20%), separate from the training and tuning processes, to avoid overfitting and ensure generalizability. For each ML algorithm, we defined comprehensive search ranges for key hyperparameters and evaluated all possible combinations to identify optimal settings. Final model performance was evaluated on a completely independent test set (20% holdout) that was not used during the hyperparameter tuning process, ensuring unbiased performance estimation. While cutoff optimization was not the primary objective, we employed the holdout validation set to determine model-specific thresholds using the Youden index. This facilitated meaningful comparisons of diagnostic metrics—including sensitivity, specificity, PPV, NPV, and accuracy—across different ML models under a binary classification framework.

### 4.4. Statistical Analyses

In the study, continuous variables were reported as means with their respective standard deviations, and categorical variables were described as counts and percentages. For the comparison of data, the Student’s *t*-test was employed for continuous variables and the Chi-square test for categorical variables. A stepwise multiple logistic regression analysis was conducted to identify significant predictors of AKI, and these results were then compared to those obtained from the ML algorithms. The statistical tests were two-sided, with a *p*-value threshold of less than 0.05 set for determining statistical significance. All statistical analyses were performed using R Statistical Software (version 4.2.0; R Foundation for Statistical Computing, Vienna, Austria).

## 5. Results

### 5.1. Baseline Characteristics and Clinical Outcomes

**Figure 1:**
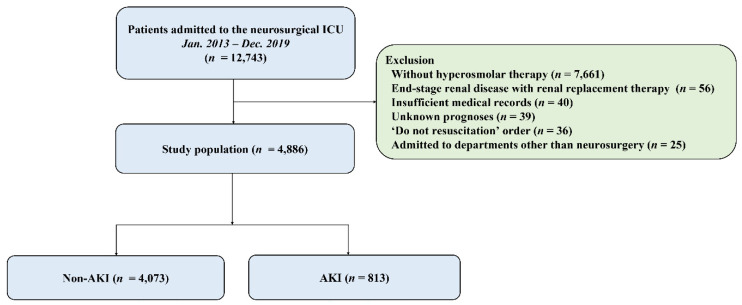
Study flow chart. ICU, intensive care unit; AKI, acute kidney injury.

This study analyzed 4886 patients (Figure 1), with a mean age of 52.0 ± 16.6 years, of whom 2044 (41.8%) were male. Malignancy (54.7%) and hypertension (29.7%) were the most common comorbidities, while brain tumor (44.9%) was the most frequent cause of ICU admission (Table 2). Patients with AKI had significantly higher rates of in-hospital mortality, 28-day mortality, ICU mortality, and ICU readmission within 48 h compared to those without AKI (all *p* < 0.001). Additionally, the length of hospital and ICU stay was longer for patients with AKI compared to those without AKI (both *p* < 0.001) (Table 3).

### 5.2. Univariate and Multivariate Logistic Analysis of Risk Factors

In the univariable analysis, significant differences were observed in most variables between the two groups, except for age, gender, malignancy, behavioral risk factors, and duration of continuous renal replacement therapy (Table 2). However, in the multivariable analysis, several factors were found to be significantly associated with AKI (C-statistic: 0.814, 95% CI: 0.797–0.814), including male gender (adjusted odds ratio [OR]: 0.70, 95% confidence interval [CI]: 0.57–0.85), hypertension (adjusted OR: 1.16, 95% CI: 1.05–1.28), intracranial hemorrhage (adjusted OR: 3.25, 95% CI: 2.31–4.56), subarachnoid hemorrhage (adjusted OR: 3.30, 95% CI: 2.31–4.68), traumatic brain injury (adjusted OR: 3.76, 95% CI: 2.57–5.48), spinal surgery (adjusted OR: 2.77, 95% CI: 1.67–4.46), central nervous system infection (adjusted OR: 5.48, 95% CI: 2.72–10.90), APACHE II score on ICU admission (adjusted OR: 1.16, 95% CI: 1.05–1.29), mechanical ventilation (adjusted OR: 1.32, 95% CI: 1.21–1.44), ICP monitoring (adjusted OR: 1.20, 95% CI: 1.11–1.29), use of vasopressor (adjusted OR: 1.18, 95% CI: 1.09–1.27), initial chloride level (adjusted OR: 5.21, 95% CI: 1.01–49.10), delta chloride (adjusted OR: 5.08, 95% CI: 1.22–35.61), and serum osmolality (adjusted OR: 1.36, 95% CI: 1.21–1.53) (Table 4).

### 5.3. Machine Learning-Based Prediction of Acute Kidney Injury

**Figure 2:**
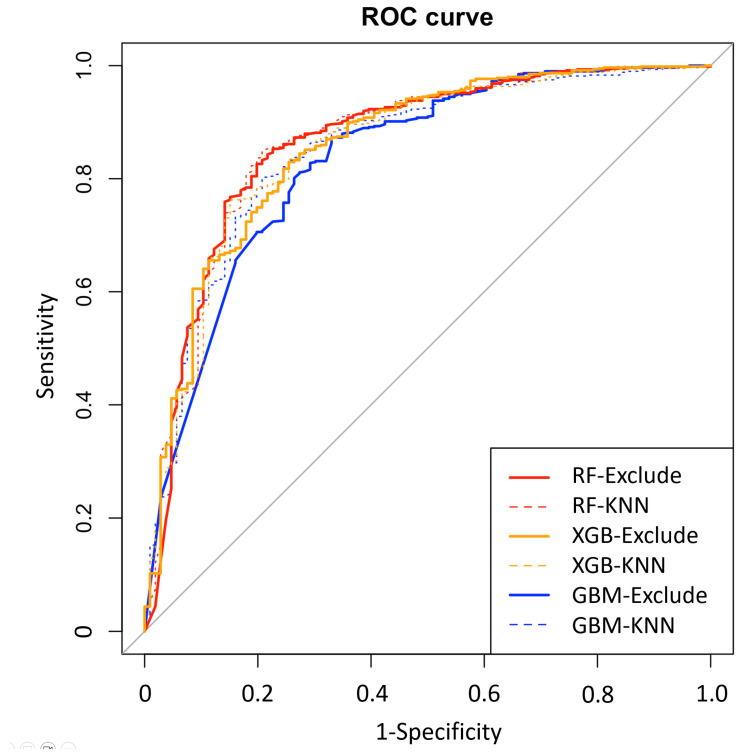
Receiver operating characteristic (ROC) curves demonstrating model generalization across different missing data handling approaches. Performance comparison of eXtreme Gradient Boosting (XGB), Gradient Boosting Machine (GBM), and Random Forest (RF) models using exclude (excluding subjects with missing data) and k-nearest neighbors (KNN) imputation approaches. The consistent performance across preprocessing methods demonstrates model robustness and generalization capability.

**Figure 3:**
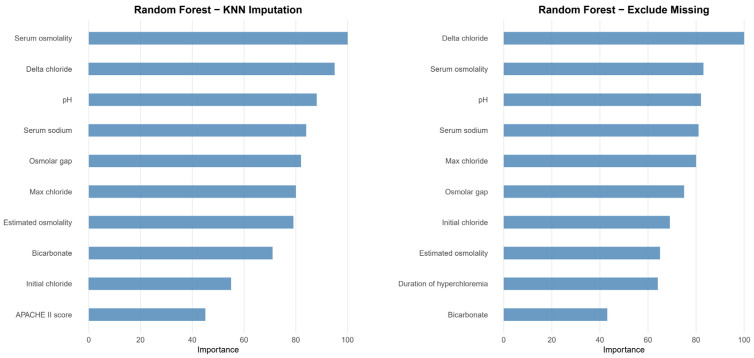
Variable importance ranking for the top 10 predictors in RF models across different preprocessing approaches. The left panel shows variable importance for RF with KNN imputation, while the right panel displays results for RF with missing data exclusion. Both preprocessing methods demonstrate consistent identification of key predictive variables, with delta chloride and serum osmolality emerging as the most important predictors. The magnitude of importance is scaled so that the maximum value is 100. The remarkable consistency in variable rankings between the two approaches demonstrates the robustness of these predictive features regardless of missing data handling methodology, supporting the clinical relevance of these biomarkers for AKI prediction in neurocritical care patients receiving hyperosmolar therapy.

After initial analysis using ML models, AdaBoost, Bagging, MARS, and SVM were excluded in the final analysis because of low predictive power (ROC < 0.78 compared to the top three algorithms achieving ROC > 0.80). Finally, we utilized the top three algorithms, XGBoost, GBM, and RF, with the highest accuracy from the 7 ML algorithms. The hyperparameter optimization process yielded 10-fold cross-validation AUROC values ranging from 0.77 to 0.89 across different algorithms, with optimal models achieving AUROC values of 0.83–0.86 on the independent test set (Table 5). Comprehensive performance evaluation of ML models across different preprocessing approaches is shown in Table 5 and Figure 2. Overall, all three models demonstrated excellent proficiency in predicting AKI, with accuracy scores ranging between 0.78 and 0.84. RF with KNN imputation achieved the highest overall performance among all ML algorithms (AUROC: 0.86, 95% CI: 0.82–0.91; sensitivity: 0.79, 95% CI: 0.70–0.87; specificity: 0.85, 95% CI: 0.82–0.88; accuracy: 0.84, 95% CI: 0.81–0.87) (Table 5). The top 10 critical features predicting AKI in the RF model across different preprocessing approaches are shown in Figure 3. The feature importance rankings remained remarkably consistent between the two preprocessing methods, demonstrating the robustness of key predictive variables regardless of the missing data handling approach. In the comparative analysis of ML models, delta chloride consistently ranked as the most significant predictor, particularly within the XGBoost model, where it was followed by variables such as pH, age, serum osmolality, bicarbonate, and osmolar gap. The models demonstrated robust generalization capability, maintaining consistent performance across different preprocessing approaches, including missing data exclusion and KNN imputation, with AUROC values consistently above 0.83 for all three algorithms. The robustness of our models was further validated through a comprehensive 10-fold cross-validation analysis (Table 6). All algorithms demonstrated consistent performance across folds, with mean AUROC values ranging from 0.824 to 0.836 and standard deviations below 0.042, indicating excellent stability and reliability. The RF exclude missing approach achieved the highest cross-validation performance (AUROC: 0.836 ± 0.035), followed closely by RF with KNN imputation (AUROC: 0.832 ± 0.041). The low variability across folds confirms the generalizability of our models and supports their potential for clinical implementation.

All performance metrics were calculated on the independent test set (20% holdout) that was not used during model training or hyperparameter tuning. The exclude missing approach used only complete cases, while KNN imputation filled missing values using the k-nearest neighbors algorithm. SMOTE was applied only to RF and XGBoost for comparison of balancing techniques.

All AUROC values were obtained through stratified 10-fold cross-validation on the training set (80% of the total dataset). The consistent performance across folds demonstrates the robustness and generalizability of all models, regardless of the preprocessing approach.

## 6. Discussion

In this study, neurocritically ill patients with AKI had significantly higher rates of in-hospital mortality, 28-day mortality, ICU mortality, and ICU readmission within 48 h when compared to those without AKI. In our investigation into the utility of ML for predicting AKI in neurocritically ill patients, we have uncovered a pivotal role for advanced analytics in the realm of neurocritical care. By deploying a suite of sophisticated ML algorithms—notably XGBoost, GBM, and RF—our study has demonstrated an improvement in predictive accuracy over traditional logistic regression methods. This superior performance underscores the potential of ML to transform current predictive models for AKI, especially in patients undergoing hyperosmolar therapy. Our findings have identified delta chloride levels as a particularly potent predictive variable, alongside other factors such as patient age, serum osmolality, bicarbonate levels, and the osmolar gap. The XGBoost model emerged as the most accurate, offering a robust tool for clinicians to anticipate and strategize against the development of AKI. Utilizing ML to identify risk factors for AKI in neurocritical care could be instrumental in reducing its incidence.

In neurocritical care, AKI stands out as a particularly critical condition due to its significant impact on patient outcomes [10]. Studies have consistently shown that AKI in this patient population is associated with increased mortality rates, extended lengths of stay in the ICU, and potential long-term neurological impairments [8]. These consequences are not just a reflection of the severity of the primary neurological condition but also indicative of the complex interplay between renal and brain function [8,10]. The development of AKI can exacerbate cerebral edema, potentially worsening the patient’s neurological status. Moreover, AKI’s impact on other organ systems can complicate the overall clinical management of these patients, often leading to a cascade of interventions that further extend hospitalization and recovery times [11]. Thus, understanding and addressing AKI in neurocritically ill patients is paramount, as its occurrence can be both a marker of severity and a contributor to adverse outcomes. This underscores the need for advanced diagnostic and therapeutic strategies aimed at early detection and effective management of AKI to improve overall patient prognosis in neurocritical care.

In the context of AKI among neurocritically ill patients, traditional predictors have included baseline renal function, illness severity, nephrotoxic drug use, hemodynamic instability, sepsis, and chronic kidney disease [7,8,10]. Our study, however, brings to light novel predictive factors such as delta chloride levels, pH imbalances, and specific serum osmolality aspects. We discovered a significant association between changes in chloride levels and AKI occurrence, a factor traditionally underemphasized. This novel finding underscores the need to pay closer attention to chloride level fluctuations, recognizing their potential as indicators of AKI risk. Hyperchloremia, which can lead to intravascular dehydration and vasoconstriction, emerges as a crucial modifiable risk factor [29]. Understanding the intricate relationship between chloride levels and kidney injury is vital. It necessitates vigilant monitoring and proactive management to mitigate AKI risks and enhance patient outcomes in neurocritical care. In addition, the occurrence of hyperchloremia-induced metabolic acidosis may also be a contributing factor to the development of AKI in neurocritically ill patients [31,40].

In this study, the application of ML algorithms has shown an interesting ability to detect complex patterns in clinical data that traditional statistical methods might overlook. The strength of ML lies in its capacity to process large datasets, revealing intricate relationships that can enhance the accuracy and effectiveness of diagnostics [41]. This is especially relevant in neurocritical care, where early and accurate detection of conditions like AKI is crucial for patient outcomes [16]. The use of ML in medical diagnostics represents a significant step forward, offering clinicians an additional tool for managing complex patient scenarios. This approach not only holds promise for improving patient care but also has the potential to streamline healthcare processes, reduce costs, and minimize medical errors [17].

Our study builds upon and extends previous valuable research in neurocritical care AKI prediction. While earlier studies have made important contributions to understanding AKI in neurocritical populations [7,14,29], opportunities remain for further advancement in several key areas. Previous research has primarily utilized traditional statistical approaches, particularly logistic regression, which have provided foundational insights into AKI risk factors [14,30,31,32]. However, the complex nature of neurocritical care settings may benefit from more sophisticated analytical approaches capable of capturing non-linear relationships and multivariable interactions. Additionally, while some studies have explored the role of electrolyte disturbances in AKI development [30,31,32], a comprehensive investigation of novel biomarkers such as delta chloride levels represents an area for continued exploration. Our study contributes to this evolving field by: (1) leveraging a substantial cohort of neurocritical care patients receiving hyperosmolar therapy, (2) systematically evaluating multiple ML algorithms to optimize predictive performance, (3) identifying and validating delta chloride as a highly predictive biomarker, and (4) developing a practical XGBoost model with robust performance for potential clinical implementation. These findings complement existing literature and represent a step forward in developing more precise and clinically applicable AKI prediction tools for neurocritical care settings.

This study has significant practical and theoretical implications for neurocritical care. From a practical standpoint, our XGBoost model can be integrated into electronic health record systems to provide real-time AKI risk assessment, enabling early identification of high-risk patients and facilitating timely nephroprotective interventions [24]. The identification of delta chloride as the most significant predictor offers clinicians a readily available biomarker for continuous monitoring, potentially reducing healthcare costs through preventive rather than reactive approaches [42]. From a theoretical perspective, our findings advance understanding of AKI pathophysiology by highlighting the critical role of chloride dynamics in kidney injury development [15]. The prominence of delta chloride provides new insights into hyperosmolar therapy-induced AKI mechanisms and supports the brain-kidney interaction paradigm [8]. Furthermore, our successful application of ML demonstrates the potential for advanced analytics to uncover previously unrecognized patterns in complex clinical datasets, contributing to precision medicine approaches in critical care [28].

The balance between sensitivity and specificity represents a critical consideration for clinical implementation of AKI prediction models. While high sensitivity is essential for early detection and prevention of AKI, adequate specificity is equally important to minimize false positive predictions that could lead to unnecessary interventions, increased healthcare costs, and alert fatigue among clinicians. Our final models demonstrate well-balanced performance with both sensitivity (79–81%) and specificity (78–85%) at clinically acceptable levels. The choice of optimal threshold should ultimately be guided by clinical context, with consideration for the relative costs of false positives versus false negatives in the specific care setting.

This study had several limitations. First, this was a nonrandomized cohort study. This design inherently carries potential biases, such as selection bias and limitations in data completeness and accuracy. Second, the study’s focus on a single-center cohort might affect the generalizability of the findings. Third, the determination of appropriate sensitivity and specificity values was guided by the Youden Index. However, it’s important to note a limitation in our findings due to the relatively low specificity observed. This aspect necessitates caution in interpreting the results, as it could affect the precision of AKI predictions. Fourth, our study did not systematically assess creatine supplementation history, which could potentially influence baseline creatinine levels, though this is unlikely to be significant in our critically ill neurosurgical population. For future research, it would be beneficial to conduct prospective studies that could validate and extend our findings. Exploring additional predictive variables, possibly through multicenter collaborations, could enhance the robustness and applicability of the ML models. This approach would not only strengthen the predictive accuracy for AKI in neurocritically ill patients but also contribute to the broader understanding and management of this complex condition.

## 7. Conclusions

This study establishes the strong association of AKI with critical outcomes such as in-hospital mortality, 28-day mortality, ICU mortality, and readmission rates in neurocritically ill patients. Leveraging ML algorithms, particularly XGBoost, we’ve demonstrated enhanced predictive capabilities for AKI in patients undergoing hyperosmolar therapy. Significantly, delta chloride levels emerged as a key predictive factor. These findings not only validate the potential of ML in refining clinical diagnostics but also pave the way for improved patient management and outcomes in neurocritical care, suggesting a promising avenue for future research in this domain.

## Figures and Tables

**Table 1:** Summary of Previous Studies on Acute Kidney Injury Prediction in Critical Care Settings.

Author/Year	Population	Sample Size	Methodology	Key Predictors	Performance (AUC)	Limitations/Gaps
Buttner et al. (2020) [7]	Neurocritical care patients	1234	Logistic regression	Age, APACHE II, mechanical ventilation	0.75	Small sample; traditional statistics; no ML comparison
Peng et al. (2023) [16]	Traumatic brain injury	892	Random Forest, SVM	GCS, age, creatinine	0.78	Limited to TBI; no hyperosmolar therapy focus
Sadan et al. (2017) [14]	Subarachnoid hemorrhage	315	Logistic regression	Hyperchloremia, fluid balance	0.69	Single condition; small cohort; basic chloride analysis
Kumar et al. (2015) [29]	Subarachnoid hemorrhage	287	Logistic regression	Hypernatremia, age	0.71	Limited predictors; no advanced analytics
Koyner et al. (2018) [25]	General ICU patients	5344	Gradient boosting	Creatinine, medications, vitals	0.84	Not neurocritical care specific; no hyperosmolar focus
Tomašev et al. (2019) [13]	General hospital patients	703,782	Deep learning	Multiple clinical variables	0.92	General population; complex model; limited interpretability
Kate et al. (2016) [26]	Hospitalized elderly	1182	Random Forest, SVM	Comorbidities, medications	0.79	Elderly-specific; no ICU focus
Rashidi & Bihorac (2020) [27]	Various populations	Review study	Multiple ML approaches	Various	Variable	Review paper; no specific neurocritical focus
Oh et al. (2019) [31]	Craniotomy patients	1456	Logistic regression	Hyperchloremia, acidosis	0.73	No ML; limited to surgical patients
Riha et al. (2017) [30]	Intracerebral hemorrhage	124	Descriptive analysis	Hyperchloremia	N/A	Small sample; no prediction model
Sigmon et al. (2020) [32]	Neurological patients	89	Descriptive analysis	Chloride load	N/A	Very small sample; no prediction focus

AUC, area under the curve; ML, machine learning; TBI, traumatic brain injury; GCS, Glasgow Coma Scale; APACHE II, Acute Physiology and Chronic Health Evaluation II; SVM, support vector machine; ICU, intensive care unit; N/A, not applicable.

**Table 2:** Baseline characteristics of patients.

Variables	Non-AKI (*n* = 4073)	AKI (*n* = 813)	*p* Value
Patient demographics			
Age (year)	52.1 ± 15.7	51.8 ± 20.8	0.710
Sex, male	1703 (41.8)	341 (41.9)	0.976
Comorbidities			
Malignancy	2241 (55.0)	433 (53.3)	0.377
Hypertension	1163 (28.6)	287 (35.3)	<0.001
Diabetes mellitus	374 (9.2)	106 (13.0)	0.001
Chronic kidney disease	74 (1.8)	46 (5.7)	<0.001
Chronic liver disease	67 (1.6)	24 (3.0)	0.018
Cardiovascular disease	51 (1.3)	24 (3.0)	0.001
Behavioral risk factors			
Current alcohol consumption	1000 (24.6)	182 (22.4)	0.203
Current smoking	450 (11.0)	90 (11.1)	0.999
Cause of ICU admission			<0.001
Brain tumor	1889 (46.4)	304 (37.4)	
Microvascular decompression	940 (23.1)	27 (3.3)	
Elective vascular surgery	663 (16.3)	72 (8.9)	
Intracerebral hemorrhage	140 (3.4)	120 (14.8)	
Subarachnoid hemorrhage	114 (2.8)	101 (12.4)	
Traumatic brain injury	102 (2.5)	101 (12.4)	
Spinal surgery	93 (2.3)	26 (3.2)	
Central nervous system infection	23 (0.6)	18 (2.2)	
Cerebral infarction	21 (0.5)	15 (1.8)	
Others	88 (2.2)	29 (3.6)	
APACHE II score on ICU admission	2.4 ± 3.8	5.2 ± 6.3	<0.001
Glasgow coma scale on ICU admission	14.7 ± 1.4	13.3 ± 3.5	<0.001
ICU management			
Mechanical ventilation	471 (11.6)	378 (46.5)	<0.001
Duration of mechanical ventilation	3.1 ± 5.2	6.5 ± 7.0	<0.001
Continuous renal replacement therapy	3 (0.1)	18 (2.2)	<0.001
Duration of renal replacement therapy	1.3 ± 0.6	4.1 ± 2.4	0.067
ICP monitoring	238 (5.8)	208 (25.6)	<0.001
Duration of ICP monitoring	5.5 ± 4.1	7.3 ± 5.9	<0.001
Use of mannitol *	3662 (89.9)	606 (74.5)	<0.001
Use of glycerin *	703 (17.3)	365 (44.9)	<0.001
Use of hypertonic saline	126 (21.2)	141 (23.9)	0.030
Use of mannitol and glycerin	292 (7.2)	158 (19.4)	<0.001
Use of vasopressors	40 (1.0)	86 (10.6)	<0.001
Laboratory data			
Serum sodium level	141.5 ± 4.3	146.2 ± 9.5	<0.001
Hypernatremia	360 (8.8)	295 (36.3)	<0.001
Duration of hyperchloremia	0.1 ± 0.8	1.0 ± 2.3	<0.001
Initial chloride level	107.29 (3.89)	108.93 (7.45)	<0.001
Maximal chloride level	107.87 (4.74)	113.03 (10.50)	<0.001
Delta chloride	0.6 ± 2.6	4.1 ± 7.5	<0.001
pH	7.43 ± 0.04	7.41 ± 0.07	<0.001
Bicarbonate level	21.34 ± 2.62	20.25 ± 3.47	<0.001
Serum osmolality	302.1 ± 11.4	315.4 ± 25.7	<0.001
Osmolar gap	4.2 ± 7.6	8.4 ± 12.3	<0.001
Serum creatinine level	0.74 ± 0.32	1.17 ± 1.38	<0.001
Estimated glomerular filtration rate	100.0 ± 23.2	82.8 ± 36.3	<0.001

Data are presented as numbers (%) or means ± standard deviations. * Some patients received more than one hyperosmolar agent. APACHE II, Acute Physiology and Chronic Health Evaluation II; ICP, intracranial pressure; ICU, intensive care unit; ICP, intracranial pressure.

**Table 3:** Clinical outcomes according to acute kidney injury (AKI).

Variables	Non-AKI (*n* = 4073)	AKI (*n* = 813)	*p* Value
AKI stage			<0.001
1		623 (76.6)	
2		135 (16.6)	
3		55 (6.8)	
Clinical outcomes			
In-hospital mortality	84 (2.1)	133 (16.4)	<0.001
28-day mortality	88 (2.2)	132 (16.2)	<0.001
ICU mortality	64 (1.6)	99 (12.2)	<0.001
ICU length of stay (hour)	39.2 ± 109.2	135.7 ± 219.2	<0.001
Hospital length of stay (day)	13.57 ± 15.7	33.9 ± 83.7	<0.001
ICU readmission within 48 h	16 (0.4)	13 (1.6)	0.003

ICU, intensive care unit.

**Table 4:** Multivariable analysis of factors associated with acute kidney injury.

Variables	Adjusted OR (95% CI)	*p* Value
Sex, male	0.70 (0.57–0.85)	<0.001
Hypertension	1.16 (1.05–1.28)	0.003
Intracranial hemorrhage	3.25 (2.31–4.56)	<0.001
Subarachnoid hemorrhage	3.30 (2.31–4.68)	<0.001
Traumatic brain injury	3.76 (2.57–5.48)	<0.001
Spinal surgery	2.77 (1.67–4.46)	<0.001
Central nerve system infection	5.48 (2.72–10.90)	<0.001
APACHE II score on ICU admission	1.16 (1.05–1.29)	0.003
Mechanical ventilation	1.32 (1.21–1.44)	<0.001
ICP monitoring	1.20 (1.11–1.29)	<0.001
Use of vasopressors	1.18 (1.09–1.27)	<0.001
Initial chloride level	5.21 (1.01–49.10)	0.005
Delta chloride	5.08 (1.22–35.61)	0.041
Bicarbonate level	0.92 (0.84–1.00)	0.057
Serum osmolality	1.36 (1.21–1.53)	<0.001

OR, odds ratio; CI, confidence interval; APACHE II Acute Physiology and Chronic Health Evaluation II; ICP, intracranial pressure; ICU, intensive care unit; ICP, intracranial pressure.

**Table 5:** Model Performance on Independent Test Set Across Different Preprocessing Approaches.

Algorithm	Preprocessing	AUROC (95% CI)	Sensitivity (95% CI)	Specificity (95% CI)	PPV (95% CI)	NPV (95% CI)	Accuracy (95% CI)
GBM	Exclude missing	0.83 (0.79–0.88)	0.74 (0.64–0.82)	0.80 (0.77–0.83)	0.40 (0.33–0.47)	0.94 (0.92–0.96)	0.79 (0.76–0.82)
GBM	KNN imputation	0.85 (0.81–0.89)	0.79 (0.70–0.87)	0.80 (0.77–0.83)	0.42 (0.35–0.49)	0.96 (0.93–0.97)	0.80 (0.77–0.83)
RF	Exclude missing	0.86 (0.82–0.91)	0.80 (0.71–0.87)	0.82 (0.79–0.85)	0.45 (0.38–0.52)	0.96 (0.94–0.97)	0.82 (0.79–0.85)
RF	KNN imputation	0.86 (0.82–0.91)	0.79 (0.70–0.87)	0.85 (0.82–0.88)	0.48 (0.41–0.56)	0.96 (0.94–0.97)	0.84 (0.81–0.87)
RF	SMOTE balancing	0.61 (0.81–0.90)	0.70 (0.60–0.78)	0.50 (0.46–0.54)	0.20 (0.16–0.24)	0.90 (0.87–0.93)	0.53 (0.49–0.57)
XGBoost	Exclude missing	0.85 (0.81–0.89)	0.81 (0.72–0.88)	0.79 (0.75–0.82)	0.41 (0.34–0.48)	0.96 (0.94–0.97)	0.79 (0.76–0.82)
XGBoost	KNN imputation	0.85 (0.81–0.89)	0.79 (0.70–0.87)	0.78 (0.74–0.81)	0.39 (0.32–0.46)	0.95 (0.93–0.97)	0.78 (0.75–0.81)
XGBoost	SMOTE balancing	0.85 (0.81–0.89)	0.76 (0.67–0.84)	0.81 (0.77–0.84)	0.41 (0.34–0.49)	0.95 (0.93–0.97)	0.80 (0.77–0.83)

AUROC, area under the receiver operating characteristic curve; CI, confidence interval; GBM, Gradient Boosting Machine; KNN, k-nearest neighbors; RF, Random Forest; XGBoost, eXtreme Gradient Boosting; PPV, positive predictive value; NPV, negative predictive value; SMOTE, Synthetic Minority Oversampling Technique.

**Table 6:** 10-Fold Cross-Validation Area Under the Receiver Operating Characteristic Curve (AUROC) Results for All Algorithm-Preprocessing Combinations.

Fold	GBM Exclude	GBM KNN Imputation	RF Exclude	RF KNN Imputation	XGBoost Exclude	XGBoost KNN Imputation
Fold 1	0.773	0.899	0.816	0.906	0.810	0.884
Fold 2	0.857	0.802	0.857	0.807	0.848	0.801
Fold 3	0.900	0.825	0.837	0.826	0.832	0.819
Fold 4	0.787	0.840	0.838	0.854	0.808	0.849
Fold 5	0.838	0.826	0.777	0.826	0.796	0.833
Fold 6	0.760	0.850	0.806	0.859	0.821	0.838
Fold 7	0.835	0.851	0.882	0.849	0.845	0.839
Fold 8	0.855	0.760	0.789	0.769	0.790	0.774
Fold 9	0.810	0.784	0.878	0.799	0.870	0.778
Fold 10	0.856	0.829	0.881	0.821	0.858	0.828
Mean ± SD	0.827 ± 0.042	0.827 ± 0.040	0.836 ± 0.035	0.832 ± 0.041	0.828 ± 0.027	0.824 ± 0.031

GBM, Gradient Boosting Machine; KNN, k-nearest neighbors; RF, Random Forest; XGBoost, eXtreme Gradient Boosting; SD, standard deviation.

## Data Availability

Regarding data availability, our data are available on the Harvard Dataverse Network (http://dx.doi.org/10.7910/DVN/PWBWI6).
